# Highly pathogenic avian influenza virus H5N1 clade 2.3.4.4b from Peru forms a monophyletic group with Chilean isolates in South America

**DOI:** 10.1038/s41598-024-54072-2

**Published:** 2024-02-13

**Authors:** Gina R. Castro-Sanguinetti, Rosa González-Veliz, Alonso Callupe-Leyva, Ana P. Apaza-Chiara, Javier Jara, Walter Silva, Eliana Icochea, Juan A. More-Bayona

**Affiliations:** 1 Laboratory of Avian Pathology, Faculty of Veterinary Medicine, Universidad Nacional Mayor de San Marcos, 15021 Lima, Peru; 2 Servicio Nacional Forestal y de Fauna Silvestre (SERFOR), Ministerio de Desarrollo Agrario y Riego (MIDAGRI), 15065 Magdalena del Mar, Peru; 3 Laboratory of Virology, Faculty of Veterinary Medicine, Universidad Nacional Mayor de San Marcos, 15021 Lima, Peru

**Keywords:** Viral reservoirs, Viral genetics, Influenza virus

## Abstract

Highly pathogenic avian Influenza virus (HPAIV) has spread in an unprecedented extent globally in recent years. Despite the large reports of cases in Asia, Europe, and North America, little is known about its circulation in South America. Here, we describe the isolation, and whole genome characterization of HPAIV obtained from sampling 26 wild bird species in Peru, representing one of the largest studies in our region following the latest HPAIV introduction in South America. Out of 147 samples analyzed, 22 were positive for detection of avian influenza virus using a qRT-PCR-based assay. Following inoculation into embryonated chicken eggs, fourteen viral isolates were obtained from which nine isolates were selected for genome characterization, based on their host relevance. Our results identified the presence of HPAIV H5N1 subtype in a highly diverse wild bird species. Phylogenetic analysis revealed that these isolates correspond to the clade 2.3.4.4b, sharing a common ancestor with North American isolates and forming a monophyletic group along with isolates from Chile. Altogether, changes at the amino acid levels compared to their closest relatives indicates the virus is evolving locally, highlighting the need for constant genomic surveillance. This data evidence the chances for spillover events increases as the virus spreads into large populations of immunologically naïve avian species and adding conditions for cross species transmission.

## Introduction

Influenza A viruses are a major threat for animal and public health due to their evolutionary properties leading to the emergence of distinct novel viruses. The underlying mechanisms for evolution in Influenza viruses rely on both viral genetics and their animal hosts factors. In terms of viral genetics, its genome comprises of single-stranded, eight negative-sense viral RNAs segments^1^. Viral genome encodes for eleven proteins including some accessory proteins^2^. These eight segments are named and presented from the largest to shortest, as follows: PB2, PB1, PA, hemagglutinin (HA), nucleoprotein (NP), neuraminidase (NA), matrix (M), non-structural (NS)^3,4^. These proteins have different functions during the viral replication process. Thus, viral genome segmentation is the basis for reassortments, and recombination as evolutionary mechanisms commonly found in influenza viruses. Furthermore, influenza viruses are known to infect a wide host range^5,6^ which is the basis for spillover events and viral adaptation to novel species^1^. Altogether, influenza viruses are of the most rapidly evolving pathogens of major concern for animal and public health^7^.

Hemagglutinin (HA) and Neuraminidase (NA) are important viral proteins playing fundamental roles in viral replication^8,9^ and are used for Influenza virus classification. HA is a relevant protein for viral entry into target cells, therefore it is the main target for neutralizing antibodies and vaccine development. On the other hand, NA is a tetrameric protein with enzymatic activity that allow viral release from infected cells following replication^10^. In addition to their relevance in viral replication, these proteins are used for viral classification purposes. Hence, there are sixteen (16) HA and nine (9) NA described to date, in wild birds belonging to the *Anseriformes* and *Charadriiformes* orders^11–13^. In addition, there is a description of two HA and NA subtypes of influenza-like viruses found in bats from Peru^14,15^.

Furthermore, avian influenza is classified into low and highly pathogenic avian influenza viruses based on the HA cleavage site and the clinical severity of infections in chickens^16^. Thus, most avian influenza viruses harboured by wild birds correspond to low pathogenicity avian influenza viruses (LPAIVs) causing asymptomatic or mild infections in poultry^4^. On the other hand, some H5 and H7 lineages are known to produce a severe infection leading to massive death when domestic birds are infected and, specially H5 subtypes, causing death in their wild bird hosts. Thus, the co-circulation of Low- and High-pathogenicity AIVs in wild bird species is a contributing factor for the emergence of novel Influenza viruses with distinctive features.

Highly pathogenic avian influenza viruses (HPAIVs) have caught the attention from scientists due to its concern in animal and public health^17^. Since the emergence of the HPAIV H5N1 GS/Gd lineage in China in 1996, the virus has evolved into ten clades and multiple subclades^18^. More recently, one of these genetic clades, such as the 2.3.4.4b clade, represents the main influenza virus type causing the current outbreak around the world^18–21^. Despite this virus characterization in multiple geographical areas, little is known about the circulation of the HPAIV H5N1 in South America.

Since 2006, we have implemented a local system for monitoring avian influenza viruses in wild birds in Peru. This has led to the detection, isolation, and characterization of multiple AIV isolates that corresponded to those LPAIVs^22–24^. Nevertheless, the detection of HPAIV H5 was reported on November 14th in 2022, following an outbreak of high mortality in pelicans of Paita, Piura (North coast of Peru) by the national authorities. Since the arrival of this HPAIV, more than 55,000 wild birds have died due to these HPAIV H5 infections and 46 outbreaks in poultry farms have been controlled. More recently, spillover events from avian into marine wildlife have been reported by the National Service of Natural Protected areas (SERNANP). Thus, our objective was the identification, isolation, and whole genome characterization of this HPAIV detected in Peru, in wild birds from Peru. Our study represents the first complete genetic description of this virus in South America in a large group of local wild bird species.

## Results

### Avian influenza virus is detected in a large group of wild bird species with clinical presentation of highly pathogenic avian influenza (HPAIV) infection

Samples analysed were obtained from oropharyngeal, cloacal and fecal swabs from animals as part of our active surveillance for influenza viruses or those showing clinical sings of HPAIV infection in a collaboration with SERFOR (Servicio Nacional Forestal y de Fauna Silvestre). In total, we evaluated 147 samples that corresponded to 26 wild bird species ranging from migratory birds, waterfowl and some local birds that are part of the natural ecosystem in the pacific coast of Peru. A large proportion of the samples analyzed corresponded to Franklin’s gulls (*Leucophaeus pipixcan*), Peruvian pelicans (*Pelecanus thagus*), Sanderlings (*Calidris alba*), American oystercatchers (*Haematopus palliates*), mallard ducks (*Anas platyrhynchos*) among others. A list of the species and number of samples evaluated is presented in Table 1. Following sample collection, PCR showed that 22 out of 147 samples (14.9%) resulted positive for avian influenza. Cts values of these samples ranged from 12 to 30 cycles out of the 45 PCR cycles protocol. Positive samples were processed for inoculation into embryonated chicken eggs for viral isolation.

**Table 1:** Sample distribution among wild bird species evaluated for HPAIV infection.

Number	Scientific name	Common name	Spanish name	Number of samples
1	*Leucophaeus pipixcan*	Franklin’s gull	Gaviota de Franklin	46
2	*Pelecanus thagus*	Peruvian pelican	Pelícano Peruano	12
3	*Calidris alba*	Sanderling	Playero arenero	13
4	*Haematopus palliatus*	American Oystercatcher	Ostrero Americano	8
5	*Anas platyrhynchos*	Mallard	Pato silvestre	11
6	*Larus belcheri*	Belcher’s gull	Gaviota peruana	4
7	*Parabuteo unicinctus*	Harris’s hawk	Gavilán acanelado	7
8	*Zenaida meloda*	West Peruvian dove	Paloma cuculí	7
9	*Larus dominicanus*	Kelp gull	Gaviota dominicana	6
10	*Zenaida auriculata*	Eared dove	Tórtola orejuda	5
11	*Phalacrocorax brasilianus*	Neotropical cormorant	Cormorán neotropical	4
12	*Phalacrocorax bougainvillii*	Guanay cormorant	Cormorán guanay	3
13	*Cathartes aura*	Turkey vulture	Gallinazo cabeza roja	4
14	*Nyctanassa violacea*	Yellow-crowned night heron	Guaco manglero	3
15	*Spheniscus humboldti*	Humboldt penguin	Pingüino de Humboldt	1
16	*Falco sparverius*	American kestrel	Cernícalo Americano	2
17	*Sula variegata*	Peruvian booby	Piquero Peruano	1
18	*Geranoaetus melanoleucus*	Black-chested buzzard-eagle	Águila mora	2
19	*Columba livia*	Domestic pigeon	Paloma castilla	1
20	*Ara macao*	Scarlet macaw	Guacamayo escarlata	1
21	*Amazona amazonica*	Orange-winged parrot	Amazona alinaranja	1
22	*Amazona ochrocephala*	Yellow-crowned parrot	Loro real amazónico	1
23	*Ramphastos cuvieri*	Cuvier’s Toucan	Tucán de Cuvier	1
24	*Pteroglossus castanotis*	Chesnut-eared aracari	Tucaneta parda	1
25	*Tyto alba*	Western barn owl	Lechuza de campanario	1
26	*Falco peregrinus*	Peregrine falcon	Halcón peregrino	1
				147

### Avian influenza virus was successfully isolated in embryonated eggs from wild bird species

Embryonated chicken eggs have shown to be the ideal support for avian influenza virus isolation^25^. Here, we inoculated the 22 qPCR-positive samples in eggs selected previously. Out of those inoculated, 14 samples had evidence of viral replication through embryo death and hemagglutination assay. These samples were obtained from Peruvian pelican (3/6), Belcher’s gull (3/4), Harris’s hawk (1/3), American kestrel (1/2), Guanay cormorant (1/1), Humboldt penguin (1/1), Franklin’s gull (1/2), Peregrine falcon (1/1), Peruvian booby (1/1) and Western barn owl (1/1). We did not obtain the viral isolate from the Chesnut-eared aracari sample that was positive by PCR. These findings were detected in a single round of inoculation. A summary of the results is presented in Table 2. Following isolation, positive samples were selected based on the frequency of wild bird species reported by the National authorities and their migratory capacity, for whole genome characterization.

**Table 2:** Positive samples isolated and genome sequenced in the current study.

Species	Positives PCR/total samples	N° of isolates/qPCR positives	Location	Collection date	Isolation	HA test	Genome sequencing
Franklin’s gull	2/46	1/2	Lurín, Lima	November 24th, 2022	+	+	N.S.*
Peruvian pelican	6/12	3/6	Puerto Viejo wetlands, Cañete	November 23rd, 2022	+	+	Complete
			Chorrillos, Lima	November 24th, 2022	+	+	Complete
			Punta Hermosa, Lima	November 24th, 2022	+	+	N.S
Belcher’s gull	3/4	3/3	Puerto Viejo wetlands, Cañete	November 24th, 2022	+	+	Complete
			Lima, downtown (Cercado)	December 5th, 2022	+	+	Incomplete
			Lima	December 12nd, 2022	+	+	Complete
Harris hawk	3/7	1/3	Chorrillos, Lima	December 12nd, 2022	+	+	N.S
Guanay cormorant	1/3	1/3	Villa el Salvador, Lima	December 12nd, 2022	+	+	Complete
Humboldt penguin	1/1	1/3	Chorrillos, Lima	December 20th, 2022	+	+	N.S
American kestrel	2/2	1/2	Carabayllo, Lima	December 7th, 2022	+	+	Complete
Western barn owl	1/1	1/1	Comas, Lima	December 20th, 2022	+	+	Incomplete
Peregrin falcon	1/1	1/1	Callao, Callao	December 22th, 2022	+	+	N.S
Peruvian booby	1/1	1/1	Isla Guáñape, La Libertad	December 24th, 2022	+	+	Complete
Chesnut-eared aracari	1/1	0/1	Villa el Salvador, Lima	December 14th, 2022	−	−	−

*Not sequenced.

### Highly pathogenic Avian influenza virus H5N1 clade 2.3.4.4b was identified and characterized in wild birds

**Figure 1:**
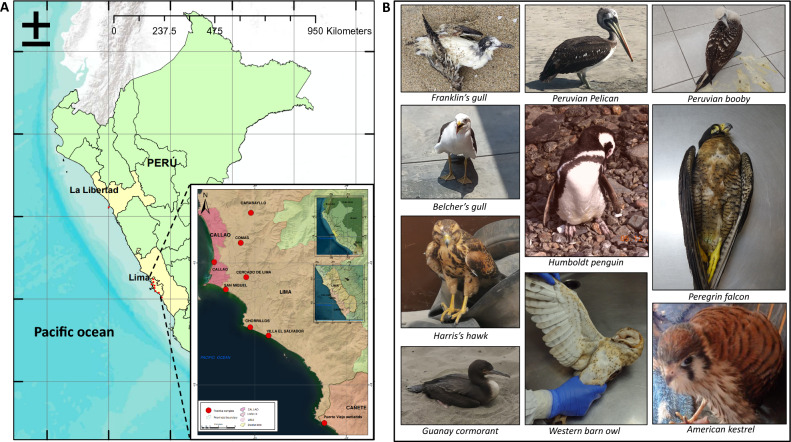
Geographical map of avian influenza isolates and species harbouring HPAIV H5N1 avian influenza virus clade 2.3.4.4b. Red dots represents the locations where isolates were obtained in Lima and La Libertad, two major cities in the coastal area in Perú (**A**). Pictures identifying the wild bird species source of the isolates characterized in our study are presented (**B**). (Geographical map was designed and prepared by Geog. José More-Bayona as requested).

**Figure 2:**
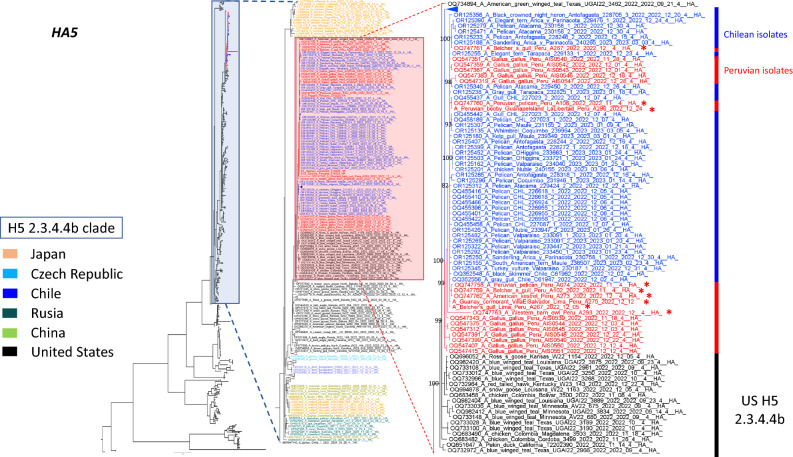
Phylogenetic analysis of H5 gene shows the virus circulating in Peru corresponds to the clade 2.3.4.4b. We collected 588 H5 complete sequences from GenBank encompassing those from Asia, Europe, and America. A phylogenetic analysis was performed using the Markov Chain Monte Carlo (MCMC) algorithm in Mr. Bayes. A general time reverse (GTR) substitution model with gamma distribution was used. Our Peruvian sequences (red asterisks) grouped together with those isolates from Chile (blue) forming a monophyletic group with a common ancestor in the US.

**Figure 3:**
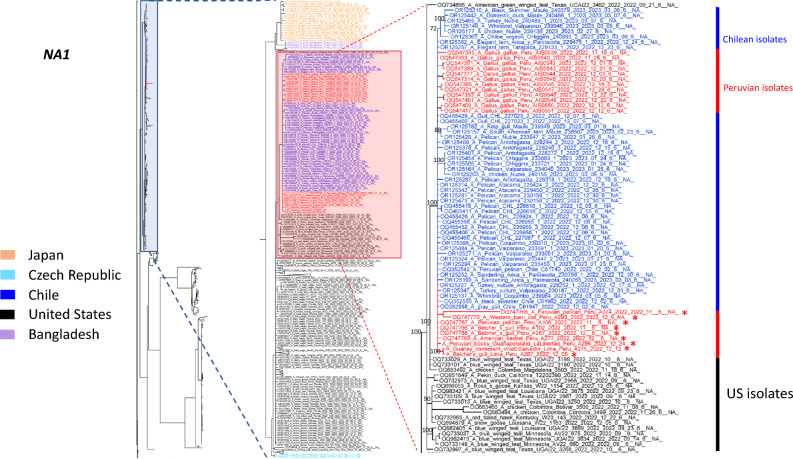
Phylogenetic analysis of N1 gene from the Peruvian isolates has a common ancestor in the US. We collected 567 N1 complete sequences from Genbank encompassing those from Asia, Europe and America. A phylogenetic analysis was performed using the Markov Chain Monte Carlo (MCMC) algorithm in Mr. Bayes. A general time reverse (GTR) substitution model with gamma distribution was used. Our Peruvian sequences (red asterisks) grouped together with those isolates from Chile (blue) forming a monophyletic groups with a common ancestor in the US.

Genetic analysis allows the subtyping of influenza viruses based on the types of HA and NA proteins. Moreover, HA amino acid sequences show differential patterns of cleavage sites to differentiate highly and low pathogenic AIVs^26^. Here, we selected 09 isolates for subtyping and genomic reconstruction. Genetic analysis at the nucleotide and amino acid levels identified that our isolates corresponded to the H5N1 subtypes. Furthermore, cleavage sites at the HA regions identified sequences corresponding to those highly pathogenic avian influenza viruses. Hence, we found the multi basic motif (PLREKRRKR↓GLF) in all highly pathogenic avian influenza viruses. A geographical map showing the sample origin distribution is shown in Fig. 1. Furthermore, we took advantage of a Bayesian model to estimate the most recent common ancestor and define the phylogenetic relationship of the H5N1 viruses identified here compared with those described in America, Asia and Europe. Based on H5 phylogenetic reconstruction, our results showed that our isolates corresponded to highly pathogenic avian influenza clade 2.3.4.4b, sharing a common ancestor with isolates obtained from snow goose in North Dakota and Kansas, in the US, 2022. Furthermore, tree topology suggested that this virus is forming a monophyletic group along with those isolated from Chile, within the 2.3.4.4b clade, and appears to accumulate changes in this protein. Phylogenetic trees of the HA and NA proteins are shown in Fig. 2 and 3.

### Genetic analysis of HA shows unique changes in the HA of HPAIV H5N1 from wild birds in Peru

Following phylogenetic reconstruction, we evaluated whether the HPAIV circulating in Peru is presenting some relevant changes compared to those members of the 2.3.4.4b. Hence, we identified some changes that were absent in those observed in most isolates from the US, while others were present in those closely related to our Peruvian isolates. Genetic distances in HA ranged from 99.08 to 99.5% in the samples sequenced. A summary of the genetic distances among all the genome segments with their closest relatives is presented in Table 3. Amino acid changes were identified in 7 out of 8 viral segments, in all nine isolates compared to their most recent ancestor. These changes suggest that our isolates along with those closely related from Chile share a common ancestor with a North American isolate, while depicting a subclade within the 2.3.4.4b phylogroup. Further studies are required to assess whether these changes are relevant for HA function or vaccine evasion. Amino acid changes observed in all nine isolates are presented in Table 4.

**Table 3:** Genetic diversity of HPAIV H5N1 compared to their closest relatives described.

Sample	Collection date	Isolate source	Ct value (PCR)	isolate	Gene	Genetic identity (%)	Reference strain origin
Sample 1	23-11-2022	Peruvian Pelican	28.7	A074	PB2	99.4	A/lesser snow goose/North Dakota/ND-10/2022
					PB1	99.54	A/lesser snow goose/North Dakota/ND-10/2022
					PA	93.52	A/lesser snow goose/North Dakota/ND-10/2022
					HA	99.29	A/snow goose/Kansas/W22-199D/2022
					NP	99.19	A/lesser snow goose/North Dakota/ND-10/2022
					NA	99.85	A/black skimmer/Chile/C61962/2022
					M	99.24	A/lesser scaup/Georgia/W22-143/2022
					NS	99.01	A/lesser snow goose/North Dakota/ND-10/2022
Sample 2	24-11-2022	Belcher´s gull	22.4	A102	PB2	99.5	A/lesser snow goose/North Dakota/ND-10/2022
					PB1	99.08	A/lesser snow goose/North Dakota/ND-10/2022
					PA	99.63	A/lesser snow goose/North Dakota/ND-10/2022
					HA	99.35	A/snow goose/Kansas/W22-199D/2022
					NP	99.19	A/lesser snow goose/North Dakota/ND-10/2022
					NA	99.86	A/black skimmer/Chile/C61962/2022
					M	99.24	A/lesser scaup/Georgia/W22-143/2022
					NS	99.3	A/lesser snow goose/North Dakota/ND-10/2022
Sample 3	24-11-2022	Peruvian pelican	26.2	A106	PB2	99.5	A/lesser snow goose/North Dakota/ND-10/2022
					PB1	99.54	A/lesser snow goose/North Dakota/ND-10/2022
					PA	99.67	A/lesser snow goose/North Dakota/ND-10/2022
					HA	99.35	A/snow goose/Kansas/W22-199D/2022
					NP	99.19	A/lesser snow goose/North Dakota/ND-10/2022
					NA	99.72	A/black skimmer/Chile/C61962/2022
					M	98.35	A/lesser scaup/Georgia/W22-143/2022
					NS	97.41	A/lesser snow goose/North Dakota/ND-10/2022
Sample 4	05-12-2022	Belcher´s gull	30.18	A267	PB2	99	A/lesser snow goose/North Dakota/ND-10/2022
					PB1	99.45	A/lesser snow goose/North Dakota/ND-10/2022
					PA	99.58	A/lesser snow goose/North Dakota/ND-10/2022
					HA	99.5	A/snow goose/Kansas/W22-199D/2022
					NP	99.12	A/lesser snow goose/North Dakota/ND-10/2022
					NA	99.77	A/black skimmer/Chile/C61962/202
					M	99.46	A/lesser scaup/Georgia/W22-143/2022
					NS	99.01	A/lesser snow goose/North Dakota/ND-10/2022
Sample 5	07-12-2022	American kestrel	16.40	A273	PB2	99.6	A/lesser snow goose/North Dakota/ND-10/2022
					PB1	99.5	A/lesser snow goose/North Dakota/ND-10/2022
					PA	99.58	A/lesser snow goose/North Dakota/ND-10/2022
					HA	99.35	A/snow goose/Kansas/W22-199D/2022
					NP	99.05	A/lesser snow goose/North Dakota/ND-10/2022
					NA	99.64	A/black skimmer/Chile/C61962/2022
					M	99.46	A/lesser scaup/Georgia/W22-143/2022
					NS	99.16	A/lesser snow goose/North Dakota/ND-10/2022
Sample 6	20-12-2022	Western barn owl	16.88	A293	PB2	99.5	A/lesser snow goose/North Dakota/ND-10/2022
					PB1	99.54	A/lesser snow goose/North Dakota/ND-10/2022
					PA	N.D	N.D
					HA	99.08	A/snow goose/Kansas/W22-199D/2022
					NP	96.76	A/lesser snow goose/North Dakota/ND-10/2022
					NA	99.86	A/black skimmer/Chile/C61962/2022
					M	N.D	N.D
					NS	N.D	N.D
Sample 7	12-12-2023	Guanay cormorant	12.52	A275	PB2	99.9	A/blue-winged teal/Texas/UGAI22-3190/2022
					PB1	99.47	A/lesser snow goose/North Dakota/ND-10/2022
					PA	99.77	A/snow goose/North Dakota/N22-06/2022
					HA	99.41	A/snow goose/Kansas/W22-199D/2022
					NP	99.18	A/lesser snow goose/North Dakota/ND-10/2022
					NA	99.79	A/black skimmer/Chile/C61962/2022
					M	99.58	A/chicken/OH/OH22-7075/2022
					NS	98.54	A/lesser snow goose/North Dakota/ND-10/2022
Sample 8	12-24-2023	Peruvian booby	23.3	A296	PB2	99.8	A/blue-winged teal/Texas/UGAI22-3190/202
					PB1	99.52	A/lesser snow goose/North Dakota/ND-10/2022
					PA	99.67	A/snow goose/North Dakota/N22-06/2022
					HA	99.35	A/snow goose/Kansas/W22-199D/2022
					NP	99.26	A/lesser snow goose/North Dakota/ND-10/2022
					NA	99.71	A/black skimmer/Chile/C61962/2022
					M	99.58	A/chicken/OH/OH22-7075/2022
					NS	98.73	A/lesser snow goose/North Dakota/ND-10/2022
Sample 9	05-12-2023	Belcher’s gull	20.9	A277	PB2	99.9	A/blue-winged teal/Texas/UGAI22-3190/2022
					PB1	99.47	A/lesser snow goose/North Dakota/ND-10/202
					PA	99.77	A/snow goose/North Dakota/N22-06/2022
					HA	99.41	A/snow goose/Kansas/W22-199D/2022
					NP	99.18	A/lesser snow goose/North Dakota/ND-10/2022
					NA	99.71	A/black skimmer/Chile/C61962/2022
					M	99.58	A/chicken/OH/OH22-7075/202
					NS	98.54	A/lesser snow goose/North Dakota/ND-10/2022

**Table 4:** Amino acid changes detected in the H5N1 influenza A viruses isolated in Peru compared to the reference genome.

Gene segment	Reference gene accession number	Reference isolate ID	Amino acid changes
HA	OP221285	A/snow goose/Kansas/W22-199D/2022	L131Q
NA	OQ352550	A/black skimmer/Chile/C61962/2022	D210G
PB2	OP377589	A/lesser snow goose/North Dakota/ND-10/2022	K249E
			R389G
PB1	OP377588	A/lesser snow goose/North Dakota/ND-10/2022	G399D
PA	OP377587	A/lesser snow goose/North Dakota/ND-10/2022	R57Q
NS	OP377586	A/lesser snow goose/North Dakota/ND-10/2022	D53G
			D139N
			G209D
			A223E
NP	OP377585	A/lesser snow goose/North Dakota/ND-10/2022	-
M	OP470767	A/lesser scaup/Georgia/W22-143/2022	N85S
			N87T
			S291R

## Discussion

Anseriformes and Charadriiformes are considered the natural reservoirs for avian influenza viruses, while migratory birds are responsible for viral spread in large geographical areas, playing a significant role in the emergence of novel viral forms. Currently, the emergence of HPAIV H5N1 clade 2.3.4.4b affecting multiple species of wild and domestic birds has been described in Asia, Europe, and America, becoming of major concern for animal and public health. Despite this, little is known about the viral circulation and genetic characterization in South America.

In Peru, the national service for animal health (SENASA) reported the first detection of a HPAIV infection in November 2022. This case was reported in a coastal city of the Northern Peru, following the occurrence of Peruvian pelicans found death in the Paita beach in Piura. Our initial findings suggested that a viral infection was responsible for causing severe disease in the animals evaluated. Our results allowed the identification of avian influenza viruses with a highly variable viral load based on the Ct values observed. Although Ct values can be used as approximation method to assess the viral load for other viral infections^27^, we did not find evidence of correlation in terms of severity and viral levels based on Ct values (data not shown). Thus, clinical presentation and the PCR results indicate that influenza virus was likely responsible for the severe cases. Further genetic characterization was required to establish the presence of a highly pathogenic avian influenza virus.

Genomic sequencing and genotyping of these viruses detected the presence of HPAIV H5N1 clade 2.3.4.4b, affecting a broad range of species. We speculate that these species play a differential role into the viral spread since some of them appeared to be more affected than others. Moreover, movement dynamics of these species might add contributing factors in viral dissemination into local areas. Despite differences in the host source, our isolates showed a high degree of genetic conservation. Our isolates belonging to a monophyletic group share a common ancestor with isolates from Chile. However, some genetic changes have been detected compared to those isolates. Hence, genetic surveillance needs to be reinforced in the upcoming months to closely track the viral evolution in our region. Evolutionary analysis shows that these viruses have been transmitted to the US from Europe and Asia and are co-circulating with other viral lineages in the Americas. It is well known, the H5N1 viruses circulating are reassortants of viruses with different origins^28^ and these events might continue in South America.

Avian influenza viruses in Peru have been tracked in wild birds since 2006 when we established a viral surveillance program that allowed the detection and characterization of only LPAIVs prior to the current outbreak. Thus, we identified that these LPAI viruses arose from common ancestors of viruses isolated in North America during the 2019 and 2022 period^22^. These findings indicate that migratory routes of wild birds play a major role into the viral entry in the South American region and are the potential route for those highly pathogenic avian influenza viruses as these birds arrive to this region in the summer period in the south hemisphere^6,29^. As expected, our current results confirmed this since these HPAIVs shared a common ancestor with those in the US.

Furthermore, although this current outbreak represents the first introduction of the HPAIV clade 2.3.4.4b virus in the Peruvian ecosystem, we speculate that multiple introductions might occur in the upcoming migration periods. Further studies are required to define other multiple introductions of the virus and whether there are alternative routes of viral entry^30^. These conditions are the fundamental basis for the emergence of viruses with novel characteristics that might infect other species including mammals. Although no human-to-human transmission has been reported, national authorities have reported the occurrence of infections in marine mammals in Peru. Even though this is not under the scope of this paper, we emphasize that genetic surveillance of these spillover events must be monitored closely since the viral adaptation to these species might reveal the capacity for infection in humans and the human-to-human transmission.

Peru is one of the most biodiverse countries around the world. Thus, the entry and spread of HPAIVs, such as H5N1 clade 2.3.4.4b, into immunologically naïve wildlife populations is rapidly diminishing the local species, with major impact in those in an endangered situation. Our study shows that multiple local avian species are affected by this virus, and some of those studied here especially Peruvian pelicans (*Pelecanus thagus*), and Guanay cormorants (*Phalacrocorax bougainvillii*) and others have been placed into an even more endangered condition. We call the authorities to pay close attention to these species of ecosystem importance. In addition, to its intrinsic importance for the local ecosystem, they do play a major economic role. The guano produced by these species is commercialized as a natural fertilizer of great value. Thus, we urge the national and regional authorities to take actions in a coordinated manner to avoid the impact on these species.

In summary, our study represents the first report of isolation and genome characterization of HPAIV H5N1clade 2.3.4.4b from the largest wild bird species pool affected of severe disease in South America. Furthermore, this report shows evidence that despite the close genetic relationship of our isolates with North American strains, there are some nucleotide and amino acid changes detected in the virus circulating in Peru, indicating the potential for spillover events into other species.

## Materials and methods

### Samples

One hundred and forty-seven samples encompassing oropharyngeal, cloacal, and fecal swabs were collected and submitted to the Laboratory of Avian Pathology at the Universidad Nacional Mayor de San Marcos in Peru, between November 2022 and January 2023. These samples included those taken as part of our active surveillance of avian influenza in Peru, along with those obtained as a research collaboration with the Servicio Nacional Forestal y de Fauna Silvestre (SERFOR). All samples were taken in a universal transport media (VTM) tube and transported to the lab during the same collection day at 4 °C and processed immediately. In brief, all swabs were resuspended in 500 µl PBS with antibiotics, filtered through 0.22-micron syringe filters to remove bacterial contamination and centrifuged for clarification (8000×*g* for 10 min). Supernatants were saved for further molecular test by a specific PCR-based assay for detection of Influenza A viruses and isolation into embryonated chicken eggs. In some instances, samples were pooled into 4–5 samples according to geographical location, species, date, etc. Some live birds samples obtained from the National Forest and wildlife Service (SERFOR), presented neurological and respiratory clinical signs suggestive of influenza infections. A summary of the samples submitted is presented in Table 1 and video recordings of representative species affected by the infection is presented in the supplementary information (Sup S8 and S9). Protocols and methods were carried out in accordance and approved by the Ethics and animal welfare committee (CEBA) by the Faculty of Veterinary Medicine in the Universidad Nacional Mayor de San Marcos (CEBA202133). We confirm that the current study adheres to the ARRIVE guidelines^31^.

### Viral detection

One hundred and forty (140) µL of samples processed were used for RNA extraction. For this purpose, we used the QIAamp Viral RNA Mini Kit (Qiagen, US), according to manufacturer’s instructions. For viral gene detection, we used the RealPCR Influenza A RNA mix for avian influenza A virus detection following the manufacturer’s instructions (IDEXX, US). The protocol, based on 45 amplification cycles, allow the identification of avian Influenza A viruses. The positive status was used as a primary criteria of sample selection for further isolation and sequencing procedures.

### Viral isolation

Viral isolation was performed by inoculation of 0.2 mL of samples into the allantoid cavity of 9–11-days-old embryonated chicken eggs in five replicates^25^. Following inoculation, eggs were incubated at 37.5 °C for 4 days and checked daily to assess embryo survival. A round of inoculation was performed to increase the viral load for improving the metagenomic sequencing of all eight genomic segments. Following inoculation, an hemagglutination test was used as screening method to evaluate the presence of an hemagglutinating agent. In brief, allantoid fluid (25 uL) was serially diluted with two-fold volume of PBS (pH 7,4). This mix was incubated into an equal proportion of 1% chicken red blood cells (RBCs). After 20 min, the bottom formation or red blood cells agglutination was recorded. Following clarification (20,000×*g* for 10 min), positive samples were selected based on wild bird species host and viral load for genome sequencing.

### Next generation sequencing

Positive samples for PCR against Influenza A virus and hemagglutination test following inoculation into embryonated eggs, were selected for genomic characterization using a metagenomic approach. First-strand cDNA was performed using the SuperScript™ III First-Strand Synthesis kit (Invitrogen™, US) with the FR20RV primer (5′-GCCGGAGCTCTGCAGATATC-3′) prior to library preparation. Library preparation was performed using Nextera XT method and sequenced on a MiSeq platform using paired 150 base read chemistry (Cambridge Technologies, Worthington, MN, USA).

### Genome assembly and annotation

Raw data (fastq files) was evaluated based on Phred score using FastQC v. 0.11.9 software^32^, while adapters were removed using Trimmomatic version 0.32^33^. The genome was assembled using SPAdes Genome Assembler version 3.13.1^34^ and annotated using Prokka genome annotation version 1.14.6^35^, along with the use of the Bacterial and Viral Bioinformatics Research Center, version 3.28.22^36^. Gene annotation was performed using the tools of the Influenza virus genome database to address all eight genome segments. Where low coverage detected the sequences were deposited as partial CD and submitted to Genbank under the accession numbers: OQ747758-OQ747763 (HA), OQ747765-OQ747770 (NA), OQ747863-OQ747868 (PB2), OQ747878-OQ747883 (PB1), OQ747873-OQ747877 (PA), OQ747895-OQ747899 (MP), OQ747884-OQ747888 (NS), OQ747889-OQ747894 (NP).

### Avian Influenza subtyping and phylogenetic analysis

**Figure 4:**
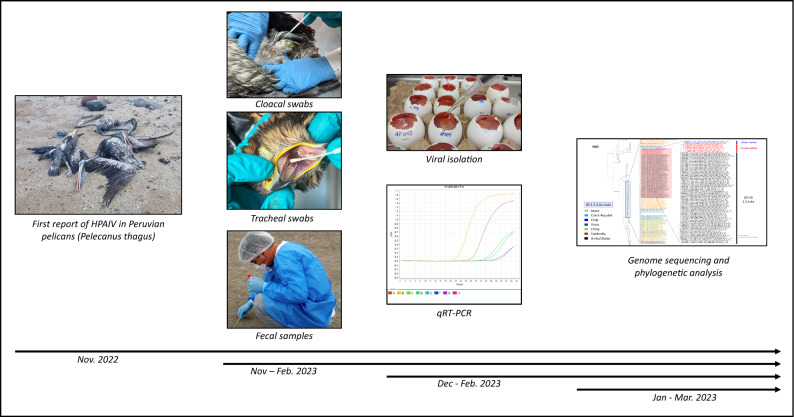
Schematic representation of the general procedure for isolation and genome characterization of HPAIV H5N1 clade 2.3.4.4b. National authorities reported the outbreak of avian influenza virus during mid November 2022. Following this report, we performed a viral surveillance along a research collaboration with SERFOR to identify the virus involved in the current outbreak. Viral isolation and detection of avian influenza virus started on December 2022 and the genome characterization was performed with those positives for the hemagglutination and qRT-PCR assays. Currently, we are continuing with the surveillance program for analysis of the HPAIV evolution in Peru.

Following genome assembly, we focused on the HA and NA subtyping. For this purpose, we collected the coding region of HA and NA genes from AIV viruses submitted to the National Center for bioinformatics information (NCBI), Influenza virus genome database (https://www.ncbi.nlm.nih.gov/genomes/FLU/Database/nph-select.cgi?go=genomeset). We only included in the analysis the coding complete sequences submitted from 2017 to 2023, isolates from avian species in Asia, Europe and America. The alignment was performed in MEGA X^37^, with posterior manual editing and files were saved as nexus files and used for phylogenetic reconstruction. We used the Markov Chain Monte Carlo (MCMC) algorithm in Mr. Bayes to estimate a Bayesian inference for phylogenetic reconstruction^38^. A general time reverse (GTR) substitution model with gamma distribution was used. Markov Chain Monte Carlo chains were run for 2 million iterations and sampled every 100 steps to allow all parameters converge with effective sample size values greater than 200, and with a burn-in of 0.25 to discard the 25% of samples at the beginning of the analysis. The consensus tree was summarized and further visualized using Figtree^39^. For the other six genome segments, sequences were retrieved from the NCBI database and selecting sequences collected from 2017 to date, for phylogenetic reconstruction, including sequences from America, Asia and Europe. A schematic representation of our methodology for detection, isolation and genome characterization is presented in Fig. 4.

### Supplementary Information

Supplementary Information 1.

Supplementary Video 1.

Supplementary Video 2.

## Data Availability

Nucleotide sequences produced in the current study have been submitted to GenBank. Here are the accession numbers for each gene segment: HA: https://www.ncbi.nlm.nih.gov/nuccore/OQ747758, https://www.ncbi.nlm.nih.gov/nuccore/OQ747759, https://www.ncbi.nlm.nih.gov/nuccore/OQ747760, https://www.ncbi.nlm.nih.gov/nuccore/OQ747761, https://www.ncbi.nlm.nih.gov/nuccore/OQ747762, https://www.ncbi.nlm.nih.gov/nuccore/OQ747763, https://www.ncbi.nlm.nih.gov/nuccore/OR591549, https://www.ncbi.nlm.nih.gov/nuccore/OR591550, https://www.ncbi.nlm.nih.gov/nuccore/OR591551. NA: https://www.ncbi.nlm.nih.gov/nuccore/OQ747765, https://www.ncbi.nlm.nih.gov/nuccore/OQ747766, https://www.ncbi.nlm.nih.gov/nuccore/OQ747767, https://www.ncbi.nlm.nih.gov/nuccore/OQ747768, https://www.ncbi.nlm.nih.gov/nuccore/OQ747769, https://www.ncbi.nlm.nih.gov/nuccore/OQ747770, https://www.ncbi.nlm.nih.gov/nuccore/OR591758, https://www.ncbi.nlm.nih.gov/nuccore/OR591759, https://www.ncbi.nlm.nih.gov/nuccore/OR591760. PB2: https://www.ncbi.nlm.nih.gov/nuccore/OQ747863, https://www.ncbi.nlm.nih.gov/nuccore/OQ747864, https://www.ncbi.nlm.nih.gov/nuccore/OQ747865, https://www.ncbi.nlm.nih.gov/nuccore/OQ747866, https://www.ncbi.nlm.nih.gov/nuccore/OQ747867, https://www.ncbi.nlm.nih.gov/nuccore/OQ747868, https://www.ncbi.nlm.nih.gov/nuccore/OR591761, https://www.ncbi.nlm.nih.gov/nuccore/OR591762, https://www.ncbi.nlm.nih.gov/nuccore/OR591763. PB1: https://www.ncbi.nlm.nih.gov/nuccore/OQ747878, https://www.ncbi.nlm.nih.gov/nuccore/OQ747879, https://www.ncbi.nlm.nih.gov/nuccore/OQ747880, https://www.ncbi.nlm.nih.gov/nuccore/OQ747881, https://www.ncbi.nlm.nih.gov/nuccore/OQ747882, https://www.ncbi.nlm.nih.gov/nuccore/OQ747883, https://www.ncbi.nlm.nih.gov/nuccore/OR591525, https://www.ncbi.nlm.nih.gov/nuccore/OR591526, https://www.ncbi.nlm.nih.gov/nuccore/OR591527. NP: https://www.ncbi.nlm.nih.gov/nuccore/OQ747889, https://www.ncbi.nlm.nih.gov/nuccore/OQ747890, https://www.ncbi.nlm.nih.gov/nuccore/OQ747891, https://www.ncbi.nlm.nih.gov/nuccore/OQ747892, https://www.ncbi.nlm.nih.gov/nuccore/OQ747893, https://www.ncbi.nlm.nih.gov/nuccore/OQ747894, https://www.ncbi.nlm.nih.gov/nuccore/OR591730, https://www.ncbi.nlm.nih.gov/nuccore/OR591731, https://www.ncbi.nlm.nih.gov/nuccore/OR591732. PA: https://www.ncbi.nlm.nih.gov/nuccore/OQ747873, https://www.ncbi.nlm.nih.gov/nuccore/OQ747874, https://www.ncbi.nlm.nih.gov/nuccore/OQ747875, https://www.ncbi.nlm.nih.gov/nuccore/OQ747876, https://www.ncbi.nlm.nih.gov/nuccore/OQ747877, https://www.ncbi.nlm.nih.gov/nuccore/OR591529, https://www.ncbi.nlm.nih.gov/nuccore/OR591530, https://www.ncbi.nlm.nih.gov/nuccore/OR591531. MP: https://www.ncbi.nlm.nih.gov/nuccore/OQ747895, https://www.ncbi.nlm.nih.gov/nuccore/OQ747896, https://www.ncbi.nlm.nih.gov/nuccore/OQ747897, https://www.ncbi.nlm.nih.gov/nuccore/OQ747898, https://www.ncbi.nlm.nih.gov/nuccore/OQ747899, https://www.ncbi.nlm.nih.gov/nuccore/OR591733, https://www.ncbi.nlm.nih.gov/nuccore/OR591734, https://www.ncbi.nlm.nih.gov/nuccore/OR591735. NS: https://www.ncbi.nlm.nih.gov/nuccore/OQ747884, https://www.ncbi.nlm.nih.gov/nuccore/OQ747885, https://www.ncbi.nlm.nih.gov/nuccore/OQ747886, https://www.ncbi.nlm.nih.gov/nuccore/OQ747887, https://www.ncbi.nlm.nih.gov/nuccore/OQ747888, https://www.ncbi.nlm.nih.gov/nuccore/OR591754, https://www.ncbi.nlm.nih.gov/nuccore/OR591755, https://www.ncbi.nlm.nih.gov/nuccore/OR591756. Additional data is available upon request.
