# Supplementary material for: Highly pathogenic avian influenza virus H5N1 clade 2.3.4.4b from Peru forms a monophyletic group with Chilean isolates in South America
Source: Sci Rep. 2024 Feb 13;14:3635. doi: 10.1038/s41598-024-54072-2 (PMC10864398; doi:10.1038/s41598-024-54072-2)
Supplement: Supplementary file 1 — Supplementary Information 1. [file 41598_2024_54072_MOESM1_ESM.pdf]

Supplementary information

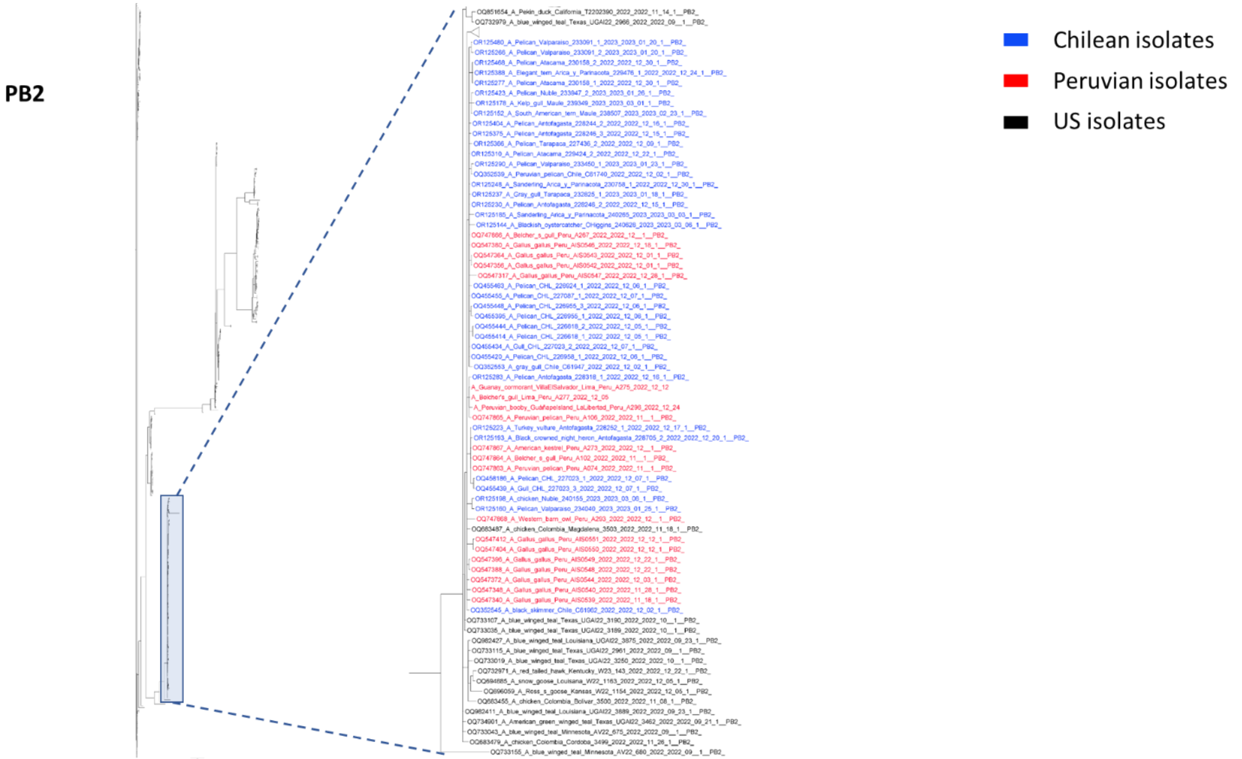

S1. ***Phylogenetic analysis of PB2 gene from the Peruvian isolates has a common ancestor in the US.*** *We collected 381 PB2 complete sequences from Genbank encompassing those from Asia, Europe and America including those sequenced in the current study. Sequences were aligned in MEGAX, while phylogenetic analysis was performed using the Markov Chain Monte Carlo (MCMC) algorithm in Mr. Bayes. A general time reverse (GTR) substitution model with gamma distribution was used. Markov Chain Monte Carlo chains were run for 2 million iterations and sampled every 100 steps to allow all parameters converge. The effective sample size values were greater than 200, and with a burn-in of 0.25 to discard the 25% of samples at the beginning. Our six Peruvian sequences grouped together with those isolates from Chile forming a subclade with a common ancestor in the US.*

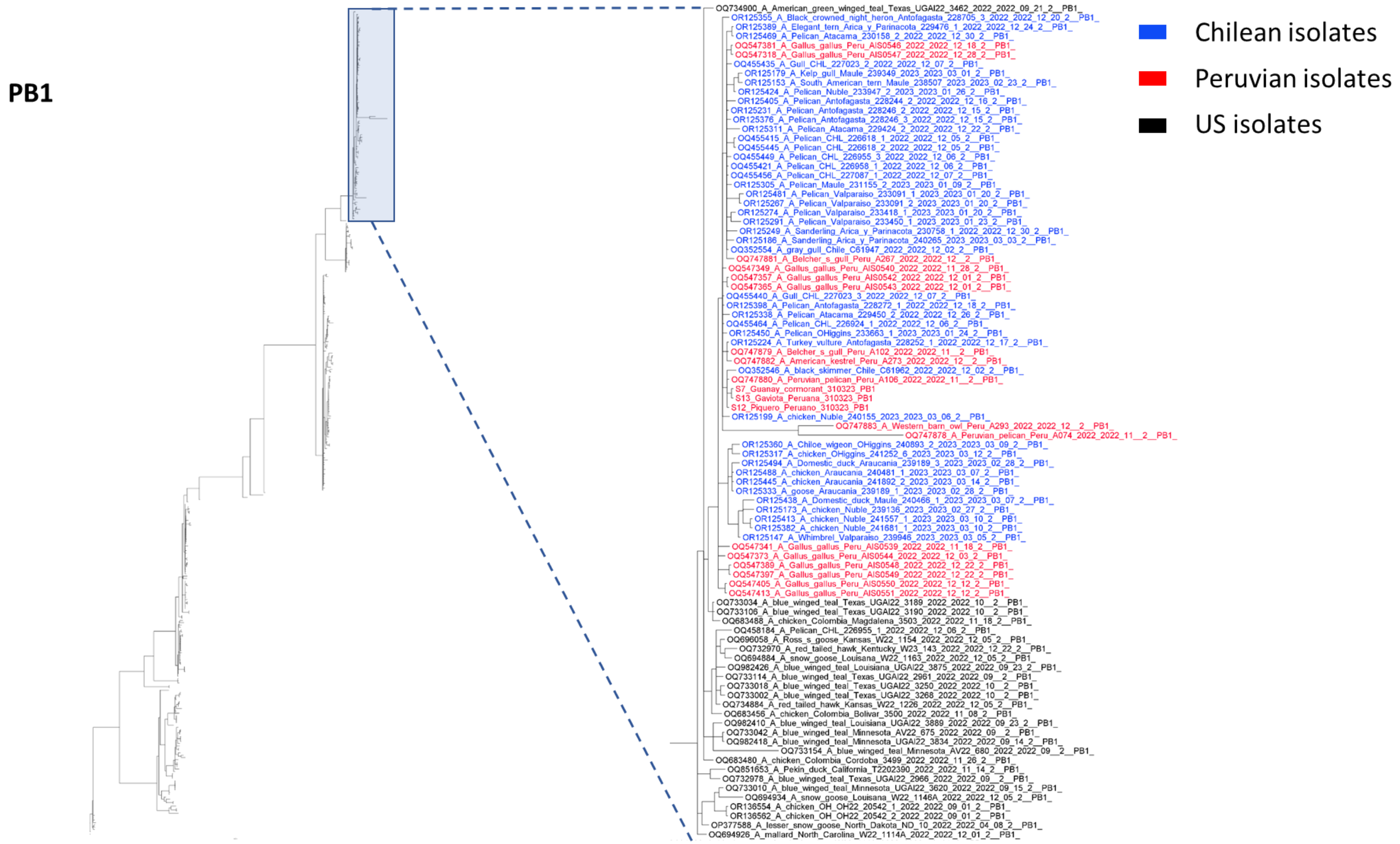

S2. Phylogenetic analysis of PB1 gene from the Peruvian isolates has a common ancestor in the US.

We collected 357 PB1 complete sequences from Genbank encompassing those from Asia, Europe and America including those sequenced in the current study. Sequences were aligned in MEGAX, while phylogenetic analysis was performed using the Markov Chain Monte Carlo (MCMC) algorithm in Mr. Bayes. A general time reverse (GTR) substitution model with gamma distribution was used. Markov Chain Monte Carlo chains were run for 2 million iterations and sampled every 100 steps to allow all parameters converge. The effective sample size values were greater than 200, and with a burn-in of 0.25 to discard the 25% of samples at the beginning. Our six Peruvian sequences grouped together with those isolates from Chile forming a subclade with a common ancestor in the US.

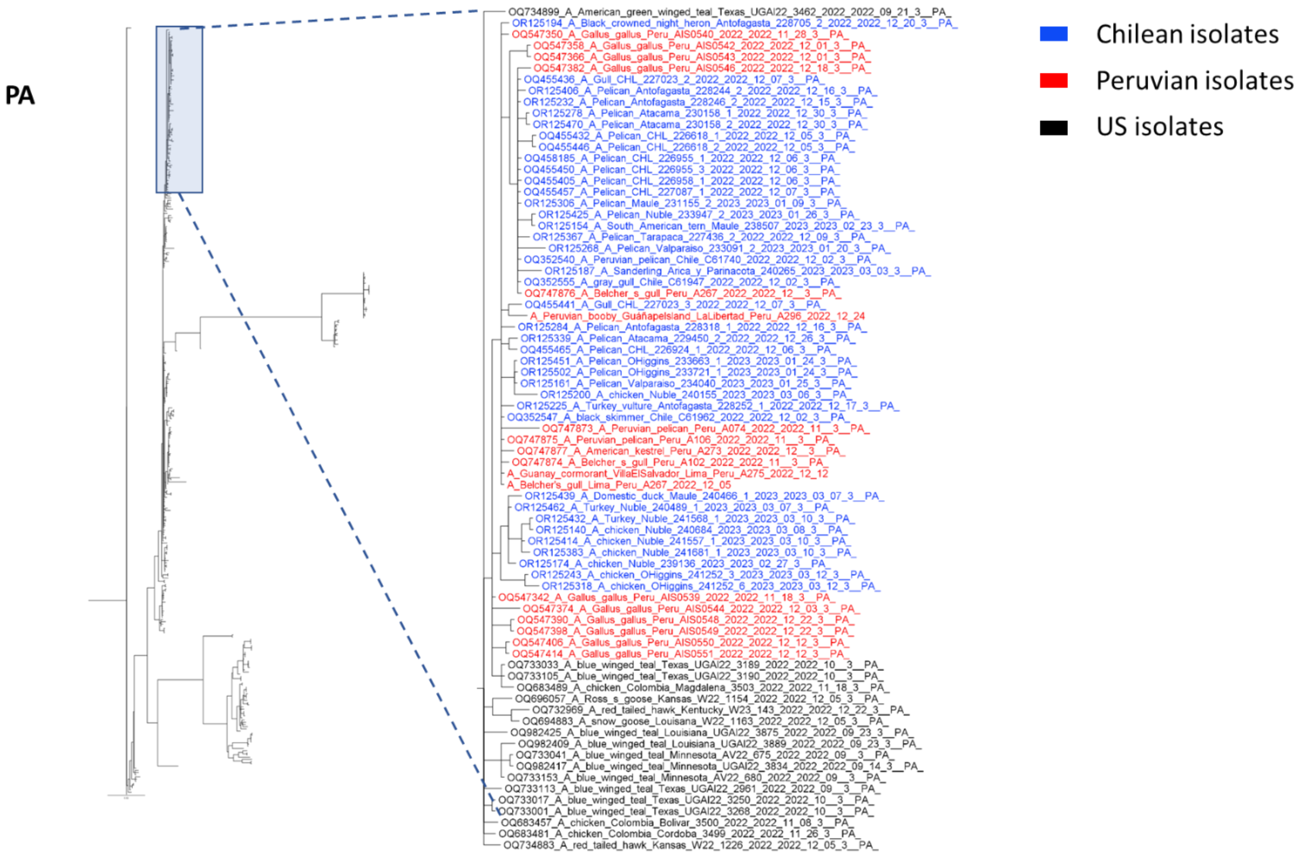

S3. Phylogenetic analysis of PA gene from the Peruvian isolates has a common ancestor in the US. *We collected 352 PA complete sequences from Genbank encompassing those from Asia, Europe and America including those sequenced in the current study. Sequences were aligned in MEGAX, while phylogenetic analysis was performed using the Markov Chain Monte Carlo (MCMC) algorithm in Mr. Bayes. A general time reverse (GTR) substitution model with gamma distribution was used. Markov Chain Monte Carlo chains were run for 2 million iterations and sampled every 100 steps to allow all parameters converge. The effective sample size values were greater than 200, and with a burn-in of 0.25 to discard the 25% of samples at the beginning. Our six Peruvian sequences grouped together with those isolates from Chile forming a subclade with a common ancestor in the US.*

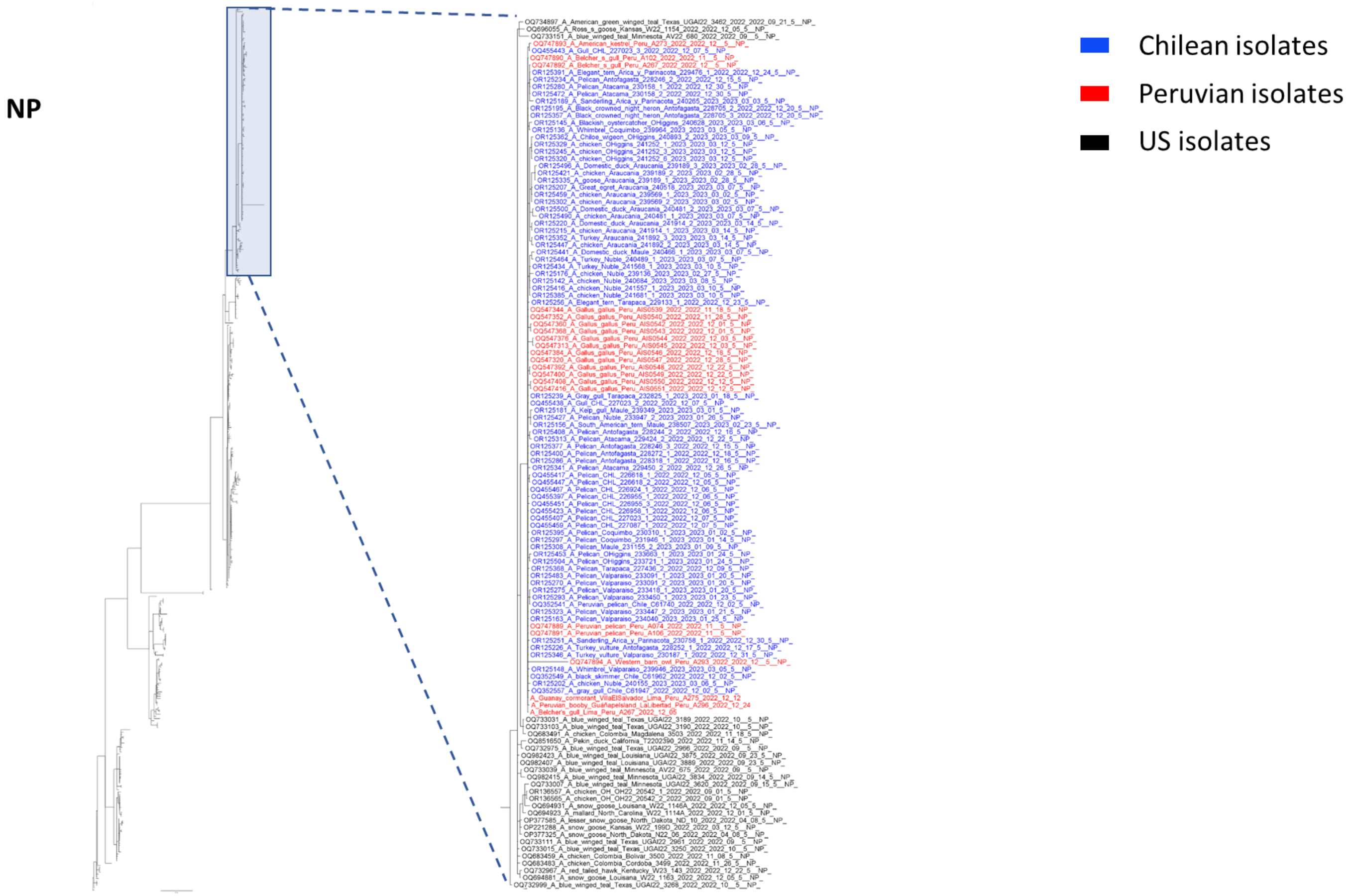

S4. Phylogenetic analysis of NP gene from the Peruvian isolates has a common ancestor in the US. *We collected 400 NP complete sequences from Genbank encompassing those from Asia, Europe and America including those sequenced in the current study. Sequences were aligned in MEGAX, while phylogenetic analysis was performed using the Markov Chain Monte Carlo (MCMC) algorithm in Mr. Bayes. A general time reverse (GTR) substitution model with gamma distribution was used. Markov Chain Monte Carlo chains were run for 2 million iterations and sampled every 100 steps to allow all parameters converge. The effective sample size values were greater than 200, and with a burn-in of 0.25 to discard the 25% of samples at the beginning. Our six Peruvian sequences grouped together with those isolates from Chile forming a subclade with a common ancestor in the US.*

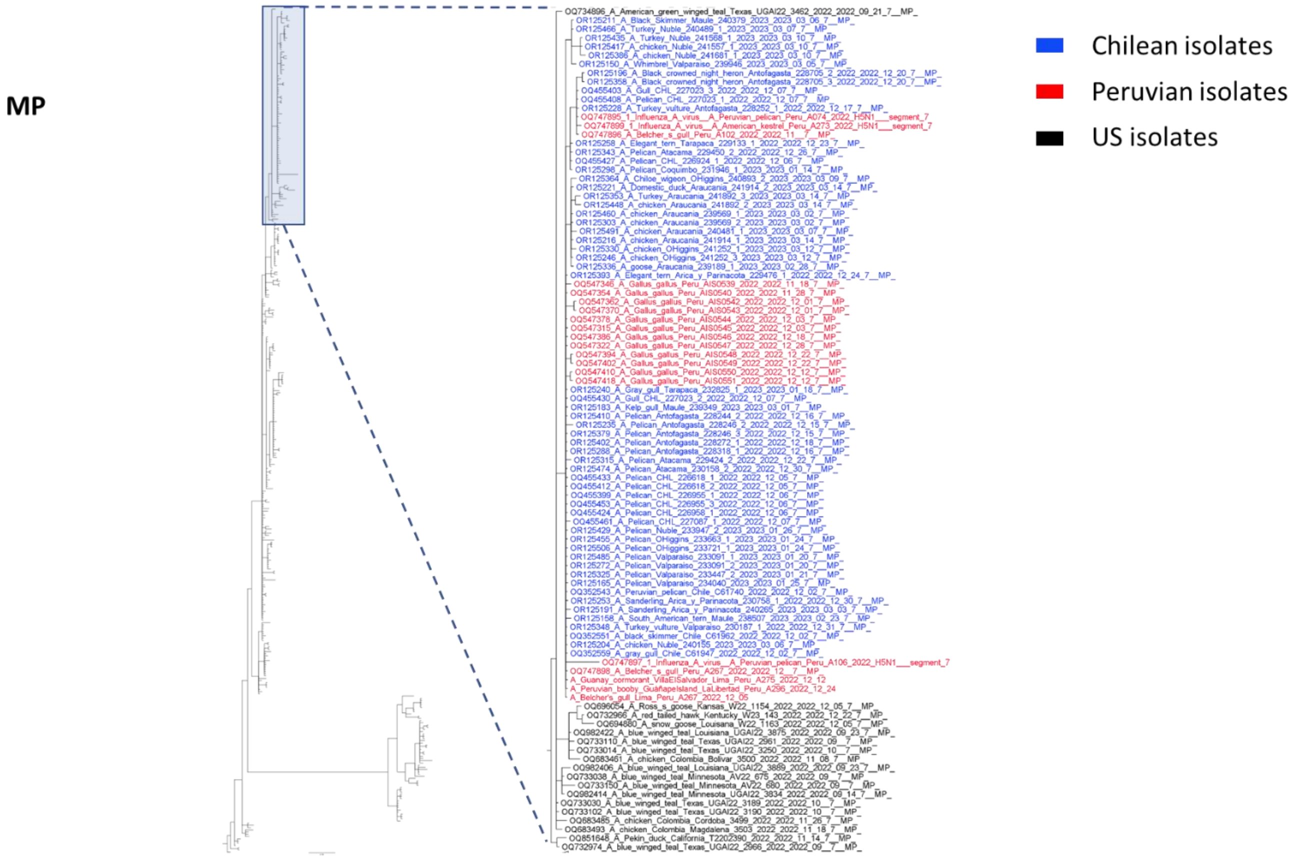

S5. Phylogenetic analysis of MP gene from the Peruvian isolates has a common ancestor in the US. *We collected 381 MP complete sequences from Genbank encompassing those from Asia, Europe and America including those sequenced in the current study. Sequences were aligned in MEGAX, while phylogenetic analysis was performed using the Markov Chain Monte Carlo (MCMC) algorithm in Mr. Bayes. A general time reverse (GTR) substitution model with gamma distribution was used. Markov Chain Monte Carlo chains were run for 2 million iterations and sampled every 100 steps to allow all parameters converge. The effective sample size values were greater than 200, and with a burn-in of 0.25 to discard the 25% of samples at the beginning. Our six Peruvian sequences grouped together with those isolates from Chile forming a subclade with a common ancestor in the US.*

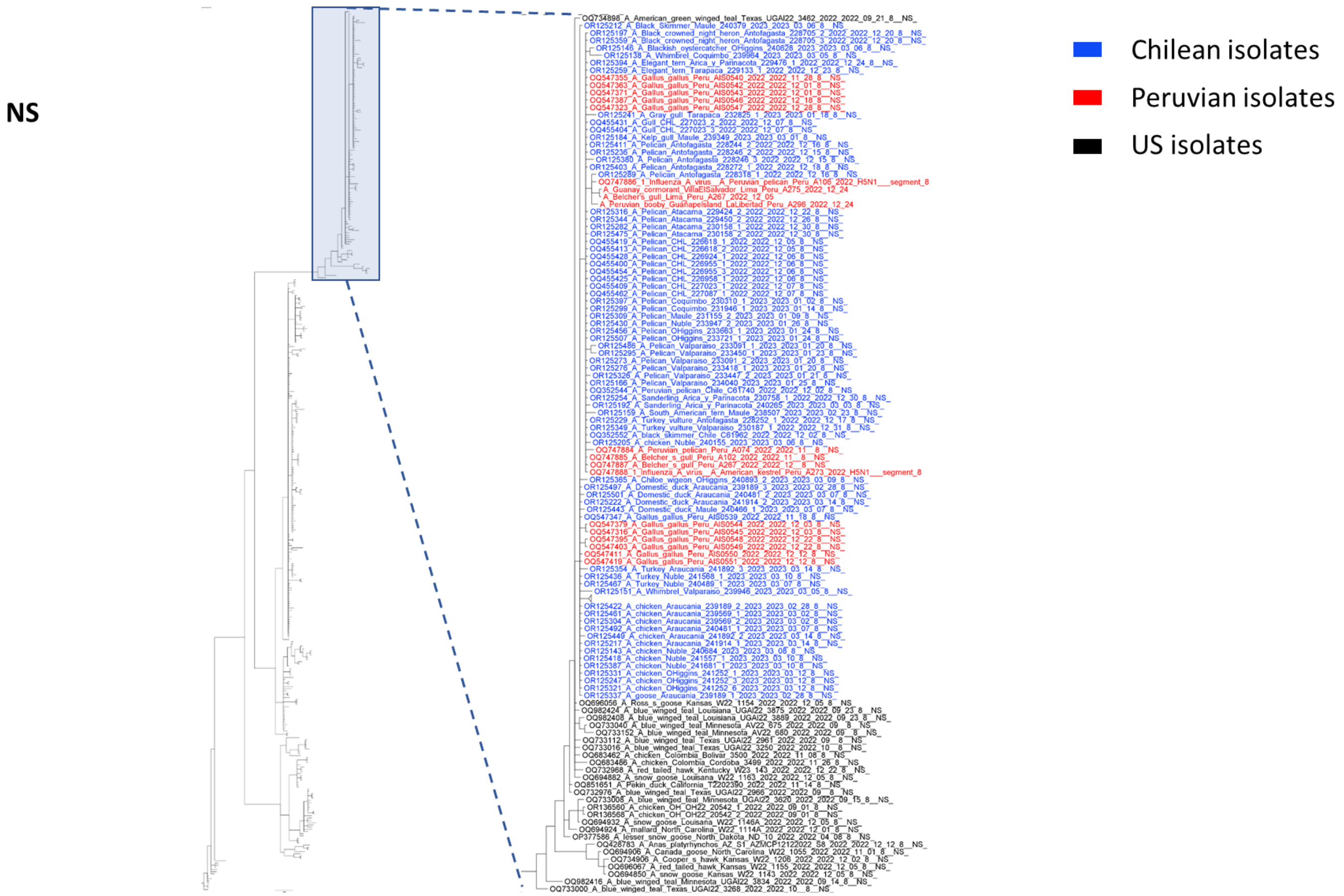

S6. Phylogenetic analysis of NS gene from the Peruvian isolates has a common ancestor in the US. *We collected 396 NS complete sequences from Genbank encompassing those from Asia, Europe and America including those sequenced in the current study. Sequences were aligned in MEGAX, while phylogenetic analysis was performed using the Markov Chain Monte Carlo (MCMC) algorithm in Mr. Bayes. A general time reverse (GTR) substitution model with gamma distribution was used. Markov Chain Monte Carlo chains were run for 2 million iterations and sampled every 100 steps to allow all parameters converge. The effective sample size values were greater than 200, and with a burn-in of 0.25 to discard the 25% of samples at the beginning. Our six Peruvian sequences grouped together with those isolates from Chile forming a subclade with a common ancestor in the US.*

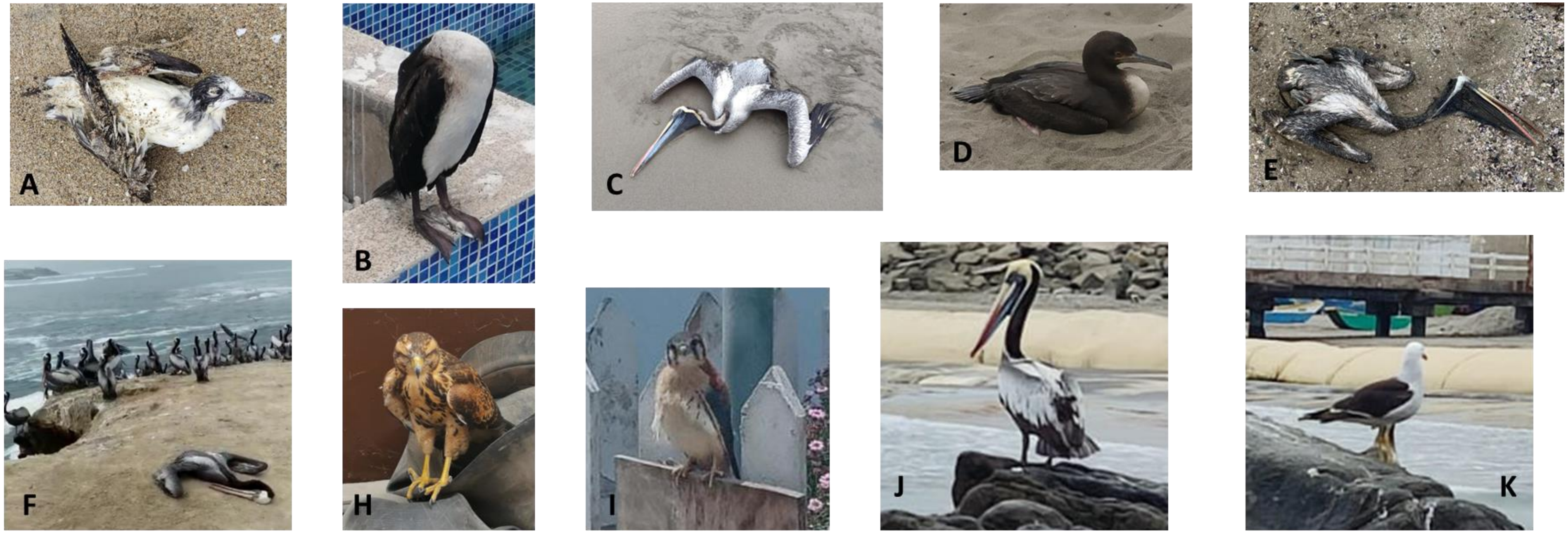

S7. Graphical pictures of some representative species affected by HPAIV H5N1 clade 2.3.4.4b infections in Peru, analysed in the current study. *Franklin's gull (A), Guanay cormorant 1 (B), Peruvian pelican 1 (C), Guanay cormorant 2 (D), Peruvian pelican 2 (E), Peruvian pelican population (F), Harris's hawk (H), American kestrel (I), Peruvian pelican 3 (J), Belcher's gull (K).*

S8. Video recording of a Peruvian pelican population showing individual cases of disease and death due to HPAIV H5N1 clade 2.3.4.4b infection in Peru.

S9. Video recording of a Belcher's gull showing neurological signs suggestive of HPAIV H5N1 clade 2.3.4.4b infection in Peru. *The isolate and genome sequence obtained in the current study (isolate A102) correspond to this individual.*
